# Longitudinal Height Growth Patterns Among Children Receiving Menaquinone-7 Supplementation

**DOI:** 10.3390/nu18121979

**Published:** 2026-06-18

**Authors:** Nghia Duc Nguyen, Hop Xuan Nguyen, Ngoc Hong Nguyen, Anh Viet Nguyen, Duong Ngoc Truong, Son Ngo Duong, Huong Thi Lan Nguyen, Long Hoang Nguyen

**Affiliations:** 1 Department of Human Anatomy, Hanoi Medical University, Hanoi 10000, Vietnam; 2 Midu MenaQ7 Joint Stock Company, Hanoi 10000, Vietnam; 3 Department of Rehabilitation, Vietlife General Clinic, Hanoi 10000, Vietnam; 4 Mirai General Clinic, Hanoi 10000, Vietnam; 5 Department of Physical Education, Hanoi Medical University, Hanoi 10000, Vietnam; 6 Faculty of Medicine, Duy Tan University, Da Nang 550000, Vietnam; 7 Institute for Global Health Innovations, Duy Tan University, Da Nang 550000, Vietnam; 8 Institute for Advanced Study in Technology, Ton Duc Thang University, Ho Chi Minh City 70000, Vietnam; 9 Faculty of Pharmacy, Ton Duc Thang University, Ho Chi Minh City 70000, Vietnam

**Keywords:** vitamin K_2_, menaquinone-7, height gain, linear growth, children, longitudinal study

## Abstract

**Background/Objectives**: Linear growth in children reflects cumulative influences of nutrition, health, and skeletal development. Vitamin K_2_, particularly menaquinone-7 (MK-7), plays an important role in bone mineralization, yet evidence regarding its potential relationship with height growth in children remains limited. This study evaluated the association between continuous MK-7 supplementation and longitudinal height growth in children. **Methods**: A longitudinal observational study was conducted among 1150 apparently healthy children aged 6–14 years in Hanoi, Vietnam (2022–2025), including 613 controls and 537 children receiving MK-7 supplementation. MK-7 was administered orally at 360 µg/day from baseline throughout follow-up. A total of 3491 repeated height measurements were collected. Analyses were stratified according to pubertal stage (no-puberty and pre-puberty). Height gain was summarized according to follow-up duration, and initial mixed-effects models were used to explore longitudinal growth trajectories. Because substantial follow-up imbalance was observed after the first follow-up assessment, the primary regression analyses were subsequently restricted to baseline and first follow-up observations. Multivariable linear regression models evaluated the interaction between MK-7 supplementation and follow-up duration after adjustment for age, sex, baseline body mass index-for-age Z-score, early sleep, and physical activity. **Results**: Height gain increased significantly with follow-up duration across all analyses (β range: 0.49–0.58 cm/month; all *p* < 0.001). MK-7 supplementation alone was not independently associated with height gain; however, positive interactions between MK-7 supplementation and follow-up duration were observed in several subgroup analyses. In the overall cohort, the interaction estimate was β = 0.05 cm/month (95% CI: 0.02–0.09). Positive interactions were observed in no-puberty children (β = 0.05; 95% CI: 0.01–0.09) and pre-puberty children (β = 0.06; 95% CI: 0.03–0.09). The largest interaction estimate was observed among pre-puberty boys (β = 0.10; 95% CI: 0.07–0.13), whereas no statistically significant interaction was observed among girls in the pre-puberty subgroup. **Conclusions**: The findings suggest that continuous MK-7 supplementation may be associated with progressively greater height gain over time rather than an immediate increase in height. The observed associations appeared more evident with longer follow-up duration, particularly among pre-pubertal boys. However, given the observational design and substantial follow-up imbalance between groups, the findings should be interpreted cautiously. Further prospective studies with balanced longitudinal follow-up are needed to clarify the potential relationship between MK-7 supplementation and pediatric linear growth.

## 1. Introduction

Child height is a widely used indicator of health, nutrition, and overall development, reflecting the complex interplay of genetic, environmental, and socioeconomic influences. Large-scale global analyses have demonstrated that mean height in children and adolescents has generally increased over recent decades, largely due to improvements in nutrition, healthcare access, and living conditions; however, substantial regional and national disparities persist [1,2]. Differences of 20 cm or more in mean height at age 19 have been reported between the tallest and shortest countries worldwide, with some regions experiencing stagnation or even declines in height gains. Height increases have been more pronounced in low- and middle-income countries, while gains in high-income countries have tended to plateau, highlighting ongoing inequalities in growth-related determinants across populations [1,2].

Vitamin K_2_ is a fat-soluble vitamin that plays essential roles in multiple physiological processes, including blood coagulation, bone metabolism, and cardiovascular health [3]. Unlike vitamin K_1_ (phylloquinone), which is predominantly involved in hepatic coagulation pathways, vitamin K_2_ (menaquinones) exhibits broader biological activity due to its higher bioavailability and wider tissue distribution [3,4]. Among the menaquinones, menaquinone-7 (MK-7) is considered the most biologically active form because of its longer half-life and sustained circulating levels, allowing more effective activation of vitamin K–dependent proteins in extrahepatic tissues [3,4,5]. The biological effects of vitamin K_2_ and MK-7 are primarily mediated through their function as cofactors for the enzyme γ-glutamyl carboxylase, which catalyzes the activation of vitamin K–dependent proteins such as osteocalcin and matrix Gla protein [4,6]. Osteocalcin is crucial for bone mineralization, while matrix Gla protein inhibits pathological vascular calcification, thereby ensuring appropriate calcium deposition in skeletal tissue rather than in blood vessels [4,6]. Through these mechanisms, MK-7 is biologically well positioned to influence skeletal growth and overall musculoskeletal development, particularly during periods of rapid growth in childhood.

Accumulating evidence suggests that vitamin K_2_ may be relevant for several aspects of pediatric health. Children are often born with relatively low vitamin K_2_ stores, and insufficient levels may negatively affect bone formation and mineral accrual during growth [4,6]. In specific pediatric populations, such as children undergoing hemodialysis, vitamin K_2_ supplementation, alone or in combination with vitamin D, has been shown to improve markers of vascular calcification, indicating potential cardiovascular benefits in high-risk groups [7]. In addition, emerging experimental and early clinical data suggest that vitamin K_2_ may exert immunomodulatory effects by reducing T-cell activation, while maintaining a favorable safety profile with no reported risk of overdose in children [4,5].

Despite these promising mechanistic and clinical observations, there is a notable gap in the literature regarding the role of MK-7 in linear growth and height development in children. To date, no well-designed clinical studies have directly evaluated the association between MK-7 supplementation and children’s height outcomes, and existing pediatric research has largely focused on broader nutritional, environmental, and psychosocial determinants of growth rather than specific micronutrients such as MK-7 [8,9,10,11]. Given the central role of bone mineralization in linear growth and the biological plausibility linking MK-7 to skeletal development, generating empirical evidence on the effects of MK-7 on child height is warranted. Therefore, the aim of this study was to evaluate the association between MK-7 supplementation and height growth in children, thereby addressing an important knowledge gap in pediatric nutrition and growth research.

## 2. Materials and Methods

### 2.1. Study Design and Participants

This retrospective longitudinal observational study was conducted at the MIDU Clinic in Hanoi from March 2022 to June 2025. The study was designed as a retrospective observational cohort reflecting real-world follow-up patterns rather than a controlled comparative intervention study. No investigator-initiated intervention or allocation was performed. Children were classified according to real-world exposure status based on caregiver decision to use or not use a commercial product containing vitamin K_2_ (menaquinone-7, MK-7). In routine clinical practice at the study site, caregivers commonly selected MK-7 supplementation for children with concerns regarding growth optimization, skeletal development, nutritional support, or overall bone health. Supplementation was initiated independently by caregivers rather than prescribed according to a standardized clinical indication or research protocol.

The study population comprised apparently healthy children aged 6–14 years at enrollment. Pubertal status was determined using Tanner staging (TS) based on clinical assessment of secondary sexual characteristics. “No puberty” was defined as Tanner stage I (TS-1). Classification was stratified by sex and age: boys ≤ 11 years and girls ≤ 10 years were considered typical TS-1; children older than these thresholds but still classified as TS-1 were identified as “delayed TS-1” [12,13,14]. For analytical purposes, “prepuberty” referred to the TS-1 group, including both typical TS-1 and delayed TS-1 children [14,15]. No participants with visible Tanner stage II (TS-2) or higher signs were included in the analytic cohort. In addition, participants who developed clinical signs consistent with TS-2 or higher during follow-up were excluded from subsequent analyses from the time of pubertal transition onward. This approach was adopted to minimize confounding from pubertal growth acceleration and to ensure that longitudinal analyses reflected growth patterns within the prepubertal biological window.

Exclusion criteria were limited to conditions with a clear potential to directly interfere with linear growth. These included a history of major surgery during the study period, congenital disorders affecting growth or bone development, chronic metabolic or endocrine diseases, and the use of medications known to influence growth or bone metabolism, such as growth hormone, long-term systemic corticosteroids, or hormonal treatments. Children taking other high-dose micronutrient supplements specifically intended to modify growth (e.g., pharmacological calcium or hormonal agents) were excluded; routine dietary intake was not restricted. Children with minor or intermediate surgical procedures and those experiencing common acute illnesses were not excluded, provided they were clinically stable at the time of assessment.

### 2.2. Sample Size, Sampling and Study Procedure

**Figure 1:**
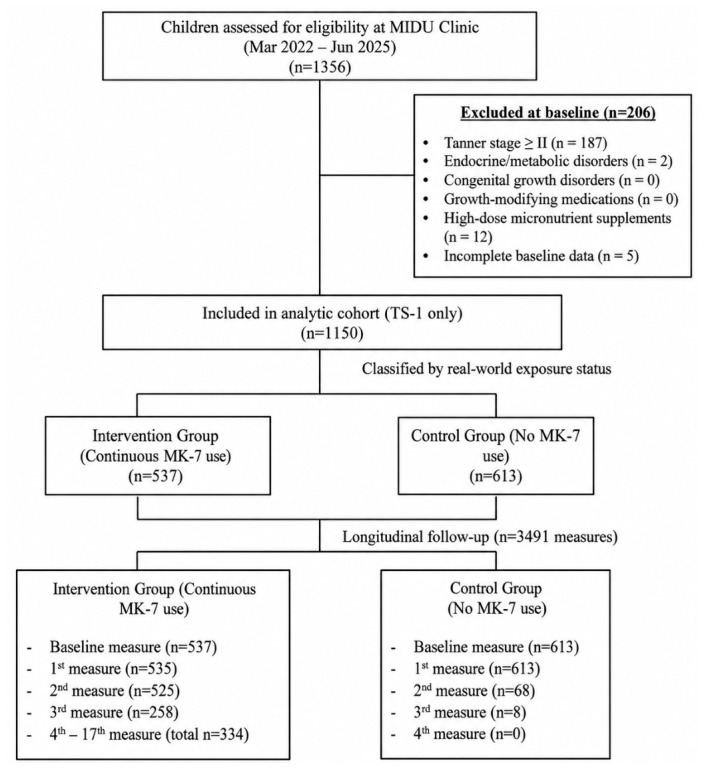
Flow chart regarding STROBE checklist. Note: Due to substantial follow-up imbalance after the first follow-up wave, regression analyses were restricted to baseline and first follow-up assessments.

A total of 1150 children were enrolled at baseline, including 613 in the control group and 537 in the MK-7 supplementation group. Group allocation was determined by caregiver choice under real-world clinical conditions; no randomization, stratification, or blinding procedures were applied. Participant enrollment, exposure classification, and longitudinal follow-up structure are presented in Figure 1.

During follow-up, 3491 anthropometric observations were collected across all visits. Baseline assessments (Time = 0) included all enrolled children, whereas 1148 observations were available at the first follow-up visit (Time = 1), comprising 613 observations in the control group and 535 in the MK-7 group. Thereafter, follow-up retention differed substantially between groups. At Time = 2, 593 observations remained available (68 control; 525 MK-7), and at Time = 3, only 266 observations were recorded (8 control; 258 MK-7). All subsequent observations from Time = 4 onward (n = 344) were derived exclusively from children continuing MK-7 supplementation.

Because follow-up attendance was determined by routine clinical practice rather than fixed study schedules, follow-up intervals varied considerably between participants. Accordingly, “Time” represented sequential visit waves rather than standardized calendar months. To improve interpretability of growth trajectories, continuous follow-up duration (months since baseline) was calculated for each observation and used to define follow-up intervals of 1–5 months, 6–12 months, and >12 months. Supplementary Table S1 summarizes the distribution of follow-up duration across sequential visit waves in both study groups. Follow-up intervals were heterogeneous and determined by real-world clinic attendance rather than fixed schedules. A progressive reduction in sample size was observed at later follow-up waves, particularly in the control group, whereas prolonged follow-up was predominantly maintained among children receiving MK-7 supplementation. Overall, the table demonstrates substantial variability in both follow-up intensity and duration across participants throughout the study period.

The marked imbalance in follow-up intensity after the first follow-up wave limited the validity of longitudinal comparisons at later visits, as these observations were derived predominantly from the MK-7 group. Therefore, regression analyses were restricted to baseline and first follow-up assessments to preserve internal comparability between groups.

### 2.3. Exposure

In this study, children in the supplementation group received vitamin K_2_ in the form of menaquinone-7 (MK-7) at a daily dose of 360 µg, administered orally from baseline throughout the follow-up period. The supplement consisted of a commercial oral formulation containing MenaQ7^®^ (vitamin K_2_ as MK-7), a naturally fermented ingredient produced using non-genetically modified *Bacillus* strains. According to the manufacturer’s technical documentation, the product contained the biologically active all-trans MK-7 form with approximately 99.8% isomeric purity and >97% overall purity. The formulation additionally included calcium glucoheptonate, magnesium gluconate, and vitamin D3 as supportive nutrients for bone health. Each 10 mL serving contained 180 µg MK-7; therefore, children received two daily servings corresponding to a total intake of 360 µg/day under caregiver supervision as part of routine nutritional supplementation. The selected dose reflected the manufacturer’s routine supplementation protocol for skeletal growth support and was biologically supported by previous studies demonstrating that MK-7 improves osteocalcin carboxylation and vitamin K status at nutritional and supplemental doses. MK-7 also possesses favorable pharmacokinetic properties, including higher bioavailability and a longer half-life than other vitamin K forms, allowing sustained activation of vitamin K–dependent proteins involved in skeletal mineralization [16,17].

The 360 µg/day dose was considered a functional supplemental dose with a broad safety margin rather than a replacement dietary requirement. Toxicological studies have demonstrated very low oral toxicity of MK-7, with no treatment-related adverse effects observed in animal studies even at doses several orders of magnitude higher than human supplementation levels, including NOAEL values up to 4500 mg/kg/day [18,19]. Furthermore, no tolerable upper intake level has been established for vitamin K because adverse effects from high oral intake have not been consistently identified in humans [20]. Pediatric guidance levels proposed for supplemental vitamin K_1_ reach approximately 370 µg/day for children aged 4–6 years and 500 µg/day for children aged 7–10 years [20], placing the dose used in this study within or near conservative pediatric supplementation ranges for school-aged children. Clinical studies further support the tolerability of MK-7 in pediatric populations. Supplementation with 45 µg/day MK-7 in healthy prepubertal children improved osteocalcin carboxylation without affecting coagulation parameters or causing adverse events [21], while pediatric hemodialysis patients safely received 100 µg/day with favorable vascular effects [7]. Emerging evidence also suggests that children commonly exhibit elevated levels of undercarboxylated osteocalcin and dephosphorylated-uncarboxylated matrix Gla protein, indicating suboptimal extrahepatic vitamin K status during growth [22,23]. Taken together, these data support the biological plausibility and safety of long-term MK-7 supplementation at 360 µg/day in children.

### 2.4. Data Collection and Measurements

Height measurement and height change (primary outcome): Standing height was the primary outcome of the study and was measured at each follow-up visit using a standardized wall-mounted stadiometer. Children were measured barefoot, standing upright with heels together, arms relaxed at the sides, and the head positioned in the Frankfurt horizontal plane. Height was recorded to the nearest 0.1 cm. All measurements were performed by trained clinicians using the same calibrated equipment to ensure consistency and minimize inter-observer variability. Repeated height measurements allowed evaluation of both absolute height and longitudinal growth trajectories over time. The primary analytical outcome was height difference from baseline (Δheight, cm), calculated as the difference between follow-up height and baseline height for each participant. Repeated measurements across follow-up visits allowed assessment of longitudinal growth trajectories and growth velocity over time.

Weight measurement and body mass index: Body weight was measured at each visit using calibrated electronic scales, with children wearing light clothing and no footwear. Weight was recorded to the nearest 0.1 kg. Body mass index (BMI) was calculated as weight in kilograms divided by height in meters squared (kg/m^2^) and was used as a key indicator of nutritional status and body composition. For each participant, body mass index-for-age Z-scores (BAZ) and height-for-age Z-scores (HAZ) were calculated using the 2007 World Health Organization (WHO) Growth Reference for children and adolescents aged 5–19 years [24]. This internationally standardized reference allows comparability across populations and provides a robust framework for evaluating both undernutrition and overnutrition in school-aged children. Nutritional status was classified according to WHO criteria. Stunting was defined as HAZ below −2 standard deviations (SD), reflecting chronic undernutrition and impaired linear growth. Thinness was defined as BAZ below −2 SD. Overweight was defined as BAZ between +1 SD and +2 SD, while obesity was defined as BAZ above +2 SD. “Any malnutrition” was defined as the presence of at least one of the following conditions: stunting, thinness, overweight, or obesity, and was used as a composite descriptive variable in baseline comparisons [25].

Demographic characteristics: Demographic information, including age (recorded in months) and sex, was collected at baseline. Pubertal stage was assessed using Tanner staging (TS) through standardized clinical evaluation. Only children classified as TS-1 were included in the main analytic cohort. Stratification distinguished typical TS-1 (boys ≤ 11 years; girls ≤ 10 years) from delayed TS-1 (older children remaining at TS-1) [12,13,14]. Age in months was used in analyses to allow precise adjustment for growth-related differences across childhood, during which growth velocity varies substantially.

Sleep habits: Sleep behavior was assessed through caregiver-reported usual bedtime. Early sleep was defined as habitual bedtime before 21:00, in accordance with age-appropriate sleep recommendations for school-aged children (9–12 h/night) [26,27]. For analysis, sleep was dichotomized as early sleep (yes/no). Sleep behavior was included as a potential confounder given its known associations with growth hormone secretion and linear growth.

Physical activity and sports participation: Information on daily participation in physical activities or sports was obtained from caregivers and clinical interviews. Regular physical activity was defined as participation in structured or moderate-to-vigorous physical activity for at least 60 min per day on ≥5 days per week, consistent with international physical activity recommendations for children [28]. The variable was coded dichotomously (yes/no). Physical activity was treated as a potential confounder because of its influence on musculoskeletal development, body composition, and overall growth.

### 2.5. Statistical Analysis

All statistical analyses were performed using Stata version 17.0 (StataCorp, College Station, TX, USA). Continuous variables were assessed for distributional characteristics using graphical methods and summary statistics. Continuous variables are primarily presented as mean ± standard deviation (SD) together with 95% confidence intervals (CIs), while categorical variables are reported as frequencies and percentages. All statistical tests were two-sided, and a *p* value < 0.05 was considered statistically significant.

Baseline characteristics of children in the control and MK-7 supplementation groups were compared separately according to pubertal subgroup to evaluate initial comparability. Pearson’s chi-square test was used for categorical variables, whereas the Kruskal–Wallis test was applied for continuous variables because of non-normal distributions. Given the non-randomized observational design, baseline differences were interpreted descriptively, and selected covariates were included in multivariable models to reduce potential confounding.

Height gain was defined as the difference between height measured at follow-up visits and baseline height. Descriptive analyses summarized mean cumulative height gain according to follow-up duration strata (1–3 months, 4–6 months, 7–9 months, 10–12 months, and >12 months). Because distributions were non-normal, between-group comparisons within each duration stratum were performed using the Mann–Whitney rank-sum test. Growth velocity was additionally calculated as cumulative height gain divided by follow-up duration (cm/month) to provide a biologically interpretable measure of longitudinal linear growth rate.

Initial analyses used linear mixed-effects models to explore longitudinal growth trajectories while accounting for repeated measurements and unequal follow-up intervals. Random intercepts were specified at the individual level to account for within-child correlation, and fixed effects included follow-up duration (months since baseline), MK-7 supplementation status, and the interaction between supplementation status and follow-up duration. Models were adjusted for age (months), sex, baseline body mass index-for-age Z-score (BAZ), early sleep behavior, and baseline physical activity.

However, during model diagnostics, residual analyses demonstrated substantial deviation from normality, including right-skewed residual distributions and influential outliers. In addition, marked differential attrition was observed after the first follow-up visit, with a substantial decline in observations within the control group at later time points. Because these conditions limited the interpretability and comparability of long-term mixed-effects analyses, the primary inferential analyses were subsequently restricted to baseline and first follow-up observations, where follow-up completeness remained comparatively balanced between groups.

Accordingly, the final regression analyses were performed using multivariable linear regression models restricted to baseline and first follow-up assessments. These models evaluated the association between MK-7 supplementation, follow-up duration, and their interaction with height gain. The interaction term was interpreted as whether continuous MK-7 supplementation modified the rate of height gain over time. Prespecified subgroup analyses were conducted according to pubertal status and sex. Effect estimates are presented as regression coefficients (β) with corresponding 95% confidence intervals (CIs).

### 2.6. Ethical Approval

The study protocol was reviewed and approved by the Ethics Committee of the Vietnam–Korea Institute of Medicine and Pharmacy Research and Training (Approval Code: 01.21/GCN-HDDDNCYSH-VKIM). Written informed consent was obtained from parents or legal guardians prior to participation, and the study was conducted in accordance with the principles of the Declaration of Helsinki.

## 3. Results

At baseline, significant differences between the control and research groups were observed only for lifestyle-related variables. In the no-puberty group, the research group had a higher prevalence of early sleep (56.0% vs. 47.1%, *p* = 0.025) and regular physical activity (61.8% vs. 42.8%, *p* < 0.001) compared with the control group. Similarly, in the pre-puberty group, early sleep (53.5% vs. 43.8%, *p* = 0.028) and physical activity (56.1% vs. 37.5%, *p* < 0.001) were significantly more common in the research group. In addition, thinness was significantly less prevalent in the research group than in the control group within the pre-puberty subgroup (1.8% vs. 6.3%, *p* = 0.012). No other baseline characteristics differed significantly between groups in either pubertal stage (Table 1).

**Figure 2:**
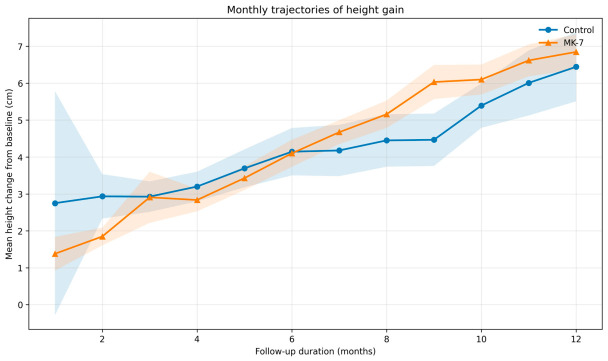
Monthly trajectories of mean height change from baseline during the first 12 months of follow-up in the control and MK-7 supplementation groups.

Figure 2 illustrates the monthly trajectories of mean height change from baseline in the control and MK-7 supplementation groups during the first 12 months of follow-up. In both groups, height gain increased progressively over time, reflecting expected longitudinal growth patterns in school-aged children. During the early follow-up period, the trajectories appeared relatively similar between groups, with substantial overlap in confidence intervals across several time points. However, from approximately months 7–9 onward, the MK-7 supplementation group appeared to show a tendency toward greater cumulative height gain compared with the control group. This separation in trajectories became more visually apparent during later months of follow-up, although confidence intervals continued to overlap at multiple time points.

Mean height gain increased progressively across longer follow-up duration groups in both the control and MK-7 supplementation groups. During the early follow-up period (1–3 months), the control group demonstrated numerically higher mean height gain compared with the MK-7 supplementation group. However, from 7 months onward, children receiving MK-7 supplementation tended to show greater height gain than those in the control group, with the difference becoming more apparent during longer follow-up durations, particularly after 12 months. The largest mean height gain was observed in the >12-month follow-up group among children receiving MK-7 supplementation. Between-group differences were statistically significant in the 1–3 months, 7–9 months, 10–12 months, and >12 months strata, whereas no significant difference was observed during the 4–6 months follow-up period (Table 2).

**Figure 3:**
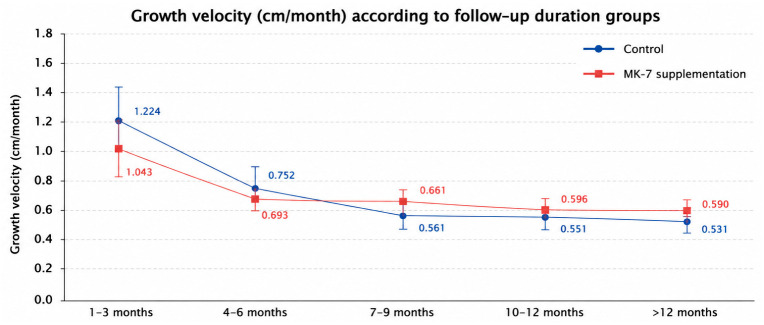
Growth velocity according to follow-up duration groups in the control and MK-7 supplementation groups.

Growth velocity decreased progressively across longer follow-up duration groups in both the control and MK-7 supplementation groups, reflecting the expected physiological decline in linear growth rate over time. During the early follow-up period (1–3 months and 4–6 months), growth velocity appeared slightly higher in the control group. However, from 7 months onward, the MK-7 supplementation group demonstrated consistently greater growth velocity compared with the control group, and this pattern persisted through the >12-month follow-up period. The difference between groups was modest but remained numerically stable at later follow-up durations, with relatively narrow confidence intervals in both groups (Figure 3).

**Figure 4:**
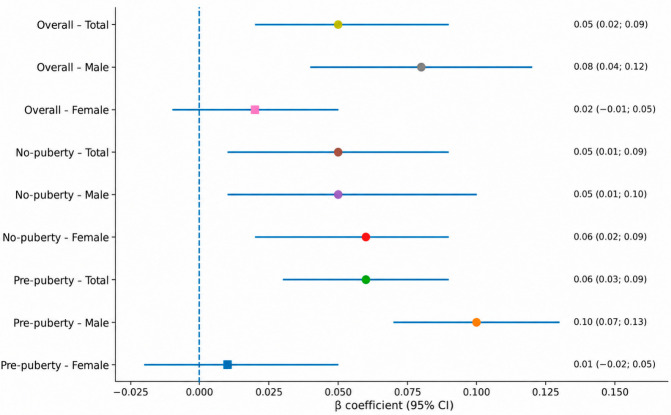
Forest plot of interaction effects between continuous MK-7 supplementation and follow-up duration on longitudinal height gain across pubertal and sex subgroups.

Regression analyses in Table 3 and Figure 4 restricted to baseline and first follow-up assessments demonstrated that follow-up duration was consistently and strongly associated with height gain across all analyses. In the overall sample, each additional month of follow-up was associated with a 0.52 cm increase in height gain (95% CI: 0.51–0.54). A significant positive interaction between continuous MK-7 supplementation and follow-up duration was observed in the total cohort (β = 0.05; 95% CI: 0.02–0.09). However, MK-7 supplementation alone was not independently associated with height gain, suggesting that the observed effect was primarily time-dependent rather than immediate.

In subgroup analyses according to pubertal status, the positive MK-7 × duration interaction remained significant in both no-puberty children (β = 0.05; 95% CI: 0.01–0.09) and pre-puberty children (β = 0.06; 95% CI: 0.03–0.09). The association appeared stronger in pre-puberty children, whereas duration itself remained a major determinant of height gain in both groups.

Gender-stratified analyses demonstrated heterogeneity in the association between MK-7 supplementation and longitudinal growth. Among boys, the interaction between MK-7 use and duration was statistically significant in both no-puberty children (β = 0.05; 95% CI: 0.01–0.10), pre-puberty children (β = 0.10; 95% CI: 0.07–0.13), and the overall male subgroup (β = 0.08; 95% CI: 0.04–0.12), indicating a progressively greater growth rate over time in boys receiving MK-7 supplementation. In contrast, no statistically significant interaction was observed among girls in either pubertal subgroup or the overall female subgroup. These post hoc subgroup analyses suggest that the time-dependent association between MK-7 supplementation and height gain may be more pronounced in boys than in girls, particularly during the pre-pubertal growth period.

## 4. Discussion

### 4.1. Discussion of Main Findings

The present study suggests that continuous MK-7 supplementation may be associated with more favorable longitudinal height trajectories compared with the control group; however, this association appeared to be primarily time-dependent rather than immediate. Across both the no-puberty and pre-puberty groups, height gain increased progressively with follow-up duration in all children, reflecting expected physiological linear growth during childhood. In the regression analyses restricted to baseline and first follow-up assessments, a positive interaction between MK-7 supplementation and follow-up duration was observed in the total cohort as well as in several subgroup analyses. These findings suggest that children receiving MK-7 supplementation tended to demonstrate a progressively greater monthly rate of height gain over time, rather than an abrupt increase in height shortly after supplementation initiation.

In no-puberty children, follow-up duration remained strongly associated with height gain in all analyses, with growth increasing by approximately 0.50–0.52 cm per month. A positive interaction between MK-7 supplementation and follow-up duration was observed in the overall no-puberty subgroup, as well as in both boys and girls. These findings may suggest that continuous MK-7 supplementation is associated with gradual differences in growth velocity over time even before the onset of pubertal acceleration. However, MK-7 supplementation alone was not independently associated with height gain, supporting the interpretation that the observed differences accumulated progressively with longer exposure duration rather than reflecting an immediate growth response.

In pre-puberty children, the association between MK-7 supplementation and longitudinal height gain appeared more heterogeneous across sex subgroups. Follow-up duration remained consistently associated with height gain in all analyses, while a positive MK-7 × duration interaction was observed in the overall pre-puberty sample and was particularly apparent among boys. Among pre-puberty boys, the interaction estimate was larger than that observed in other subgroups, whereas no statistically significant interaction was observed among pre-puberty girls. These findings may indicate that the association between MK-7 supplementation and longitudinal growth differs according to sex during the later pre-pubertal growth period. Nevertheless, because MK-7 supplementation alone was not independently associated with height gain in any subgroup, the findings are more consistent with a gradual and cumulative pattern over time rather than an early or short-term growth response.

This temporal pattern is biologically plausible. Linear growth depends on sustained processes of bone modeling, mineralization, and endochondral ossification, which require continuous activation of vitamin K–dependent proteins rather than transient exposure. Vitamin K_2_, particularly menaquinone-7 (MK-7), serves as a cofactor for γ-glutamyl carboxylase, enabling the activation of osteocalcin and other proteins involved in calcium deposition within the bone matrix [3,4]. Adequate carboxylation of osteocalcin is considered important during periods of rapid growth, when bone turnover and mineral demands are high. Experimental and clinical evidence has suggested that insufficient vitamin K_2_ status may impair osteogenic differentiation and bone formation, thereby potentially influencing linear growth over time [3,4].

In this study, MK-7 was administered continuously at a daily dose of 360 μg, which has generally been reported to be safe and well tolerated in both pediatric and adult populations, without evidence of serious adverse effects [29]. The descriptive analyses demonstrated that differences in cumulative height gain between groups became more apparent during longer follow-up periods, particularly beyond 7–12 months. This observation is broadly consistent with previous studies suggesting that the skeletal effects of MK-7 may require prolonged exposure before measurable anthropometric differences become apparent [5,30]. Endogenous production of menaquinones by the gut microbiota may be insufficient during growth, potentially supporting the importance of sustained dietary or supplemental intake in school-aged children [5,30].

The associations observed in this study should also be interpreted within the broader literature on micronutrient interventions and linear growth. Large trials and meta-analyses have generally shown that single-vitamin supplementation, such as vitamin D or vitamin A, has limited effects on height or stunting in children outside severely deficient populations, whereas zinc and multimicronutrient interventions tend to demonstrate only modest benefits [31,32,33,34,35,36]. Lipid-based nutrient supplements have shown somewhat greater effects, particularly among undernourished populations [37,38,39]. In the present study, MK-7 supplementation alone was not independently associated with greater height gain; instead, the observed associations emerged primarily through the interaction between supplementation status and follow-up duration. This pattern may be biologically plausible given vitamin K_2_’s proposed role in skeletal mineralization through activation of osteocalcin and related bone proteins [3,4]. Accordingly, MK-7 may be more relevant to gradual modulation of physiological growth processes over time rather than rapid catch-up growth.

The subgroup findings may further suggest that the observed associations vary according to sex and developmental stage. The most consistent interaction estimates were observed among boys, particularly in the pre-puberty subgroup, whereas estimates among girls were smaller and statistically non-significant. One possible explanation is that sex-related differences in skeletal maturation, hormonal regulation, growth velocity, and timing of pubertal transition could influence responsiveness to nutritional factors affecting bone metabolism [40,41]. However, because the study was observational and was not specifically designed to evaluate sex-related biological differences, these subgroup findings should be interpreted cautiously and considered exploratory. To our knowledge, previous studies have not directly evaluated MK-7 supplementation according to sex-specific longitudinal growth trajectories in children.

Taken together, the findings of the present study may suggest that MK-7 supplementation is associated with gradual differences in growth velocity over time rather than immediate changes in height. The observed time-dependent pattern may reflect a potential supportive role of MK-7 in physiological skeletal growth processes during childhood. However, given the observational design and the important limitations related to follow-up imbalance and residual confounding, these findings should be interpreted cautiously and should not be considered evidence of causality.

### 4.2. Implications

From a public health perspective, the findings suggest that any potential association between MK-7 supplementation and linear growth may be time-dependent, emerging gradually over prolonged follow-up rather than through immediate increases in height. The observed interactions between MK-7 supplementation and follow-up duration indicate that sustained exposure may be necessary before measurable differences in growth trajectories become apparent. Consequently, short-term supplementation programs may underestimate potential longitudinal associations.

The results further suggest that the observed associations may be more apparent during the pre-pubertal growth period and among boys. In these groups, children receiving MK-7 supplementation tended to demonstrate progressively greater height gain as follow-up duration increased. These findings may support the importance of considering developmental stage and sufficient follow-up duration when evaluating nutritional factors potentially related to skeletal growth.

From a programmatic standpoint, MK-7 supplementation may be considered a potential supportive component within broader child health and nutrition strategies rather than a stand-alone growth intervention. The time-dependent nature of the observed associations underscores the importance of repeated anthropometric assessments and adequate follow-up duration when studying growth-related nutritional exposures. Without prolonged observation, potential differences in longitudinal growth trajectories may remain difficult to detect.

Given the observational nature of the study, the findings should be interpreted as exploratory associations rather than causal effects. Although the analyses were restricted to baseline and first follow-up assessments to improve comparability between groups, residual confounding and selection-related differences cannot be excluded. Additional prospective studies with more balanced follow-up structures and comprehensive assessment of growth-related determinants are needed to further clarify the potential relationship between MK-7 supplementation and pediatric linear growth.

### 4.3. Limitations

Several limitations should be acknowledged when interpreting these findings. First, although most baseline anthropometric and demographic characteristics were comparable between groups, significant differences in lifestyle-related factors, specifically early sleep and physical activity, were observed at baseline, together with a lower prevalence of thinness in the pre-puberty MK-7 supplementation group. Although these variables were adjusted for in multivariable regression analyses, residual confounding cannot be excluded because of the observational and non-randomized nature of the study. In addition, several behavioral variables were dichotomized for analytical and clinical interpretability, which may have reduced variability and statistical precision.

Second, substantial differential follow-up occurred between groups over time, particularly after the first follow-up visit, where the number of observations in the control group declined markedly. This imbalance limited the validity of long-term comparative longitudinal analyses because later observations were derived predominantly from children receiving MK-7 supplementation. To minimize this source of bias and improve internal comparability, the primary inferential regression analyses were restricted to baseline and first follow-up assessments, where follow-up completeness remained relatively balanced between groups. Consequently, the findings should primarily be interpreted as short-term observational associations rather than evidence of long-term comparative effectiveness or causality.

Third, although regression models demonstrated significant interaction effects between MK-7 supplementation and follow-up duration in several subgroups, the observational design precludes definitive causal inference. The identified associations may partly reflect unmeasured differences in health behaviors, caregiver practices, nutritional support, or healthcare utilization between children who continued supplementation and those who did not.

Fourth, subgroup analyses, particularly among children with stunting or thinness, were based on relatively small sample sizes, which may have reduced statistical power and increased uncertainty around subgroup-specific estimates. Accordingly, non-significant findings in nutritionally compromised subgroups should be interpreted cautiously and not considered evidence of absence of association.

Finally, several potentially important determinants of linear growth, including detailed dietary intake, socioeconomic status, parental height, genetic influences, and more comprehensive pubertal assessments, were not available in the dataset. These unmeasured factors may have contributed to inter-individual variability in growth trajectories and may partly explain heterogeneity in the observed associations between MK-7 supplementation and height gain.

## 5. Conclusions

In conclusion, the findings of this study suggest that continuous MK-7 supplementation may be associated with progressively greater height gain over time rather than an immediate increase in height. Height gain remained strongly associated with follow-up duration and baseline characteristics across analyses, while the observed differences between groups appeared to become more evident with longer exposure duration, particularly among pre-pubertal children and boys. These findings also suggest that prolonged follow-up may be important when evaluating potential relationships between MK-7 supplementation and linear growth, as differences in growth trajectories were less apparent during earlier follow-up periods. However, given the observational design, substantial follow-up imbalance between groups, and the possibility of residual confounding, the findings should be interpreted cautiously and not considered evidence of causality. Further prospective studies with more balanced longitudinal follow-up and comprehensive assessment of growth-related factors are needed to further clarify the potential relationship between MK-7 supplementation and pediatric linear growth.

## Figures and Tables

**Table 1:** Baseline characteristics of study participants according to MK-7 supplementation status.

Variables	Category	No Puberty		*p*	Pre-Puberty		*p*
		Control (n = 325)	Research (n = 309)		Control (n = 288)	Research (n = 228)	
Sex, N (%)	Male	222 (68.3)	192 (62.1)	0.103	118 (41.0)	110 (48.3)	0.098
	Female	103 (31.7)	117 (37.9)		170 (59.0)	118 (51.7)	
Age group, N (%)	6–10 years	325 (100.0)	309 (100.0)		104 (36.1)	86 (37.7)	0.707
	11–14 years				184 (63.9)	142 (62.3)	
Stunting, N (%)	No	307 (94.5)	295 (95.5)	0.562	268 (93.1)	218 (95.6)	0.217
	Yes	18 (5.5)	14 (4.5)		20 (6.9)	10 (4.4)	
Thinness, N (%)	No	318 (97.8)	303 (98.1)	0.851	270 (93.8)	224 (98.2)	0.012
	Yes	7 (2.2)	6 (1.9)		18 (6.3)	4 (1.8)	
Overweight/obesity, N (%)	No	269 (82.8)	267 (86.4)	0.205	261 (90.6)	198 (86.8)	0.173
	Yes	56 (17.2)	42 (13.6)		27 (9.4)	30 (13.2)	
Any malnutrition, N (%)	No	246 (75.7)	249 (80.6)	0.137	226 (78.5)	184 (80.7)	0.534
	Yes	79 (24.3)	60 (19.4)		62 (21.5)	44 (19.3)	
Early sleep, N (%)	No	172 (52.9)	136 (44.0)	0.025	162 (56.3)	106 (46.5)	0.028
	Yes	153 (47.1)	173 (56.0)		126 (43.8)	122 (53.5)	
Physical activity, N (%)	No	186 (57.2)	118 (38.2)	<0.001	180 (62.5)	100 (43.9)	<0.001
	Yes	139 (42.8)	191 (61.8)		108 (37.5)	128 (56.1)	
Age (months)	Mean ± SD	94.0 ± 17.1	93.4 ± 17.4	0.604	130.4 ± 15.4	130.2 ± 16.0	0.742
Height (cm)	Mean ± SD	124.2 ± 10.1	123.8 ± 10.5	0.596	141.4 ± 9.8	141.8 ± 9.2	0.528
Weight (kg)	Mean ± SD	27.1 ± 8.1	26.4 ± 8.2	0.131	36.8 ± 10.1	37.5 ± 9.0	0.288
BAZ	Mean ± SD	0.6 ± 1.4	0.4 ± 1.4	0.088	0.2 ± 1.4	0.4 ± 1.2	0.188
HAZ	Mean ± SD	−0.3 ± 1.1	−0.3 ± 1.0	0.891	−0.3 ± 1.1	−0.2 ± 1.0	0.191

Abbreviations: MK-7, menaquinone-7; BAZ, body mass index-for-age Z-score; HAZ, height-for-age Z-score; SD, standard deviation. Stunting was defined as HAZ < −2 SD, thinness as BAZ < −2 SD, and overweight/obesity as BAZ > +1 SD according to the WHO 2007 Growth Reference. Any malnutrition indicates the presence of stunting, thinness, overweight, or obesity. Early sleep was defined as bedtime before 21:00. Physical activity was defined as ≥60 min/day on ≥5 days/week.

**Table 2:** Height gain according to follow-up duration strata in the control and MK-7 supplementation groups.

Follow-Up Duration	Group	n	Mean (cm)	SD	95% CI	*p*-Value *
1–3 months	Control	92	2.92	1.69	2.58–3.27	<0.001
	MK-7 supplementation	225	2.26	2.77	1.89–2.62	
4–6 months	Control	162	3.72	1.98	3.41–4.03	0.248
	MK-7 supplementation	312	3.43	1.79	3.23–3.63	
7–9 months	Control	124	4.38	2.31	3.97–4.79	<0.001
	MK-7 supplementation	305	5.26	2.03	5.03–5.48	
10–12 months	Control	98	6.01	2.27	5.56–6.46	0.023
	MK-7 supplementation	277	6.51	2.17	6.26–6.77	
>12 months	Control	204	8.03	3.92	7.49–8.57	<0.001
	MK-7 supplementation	507	9.88	4.44	9.50–10.27	

* Mann–Whitney rank-sum test comparing height gain between groups within each follow-up duration stratum.

**Table 3:** Regression results for height gain between baseline and first follow-up assessments.

Variable	Total (n = 2298)	Gender	
		Male (n = 1315)	Female (n = 983)
	β (95% CI)	β (95% CI)	β (95% CI)
**No-puberty children (n = 1094)**			
Using MK-7 continuously × Duration of follow-up (months)	0.05 * (0.01; 0.09)	0.05 * (0.01; 0.10)	0.06 * (0.02; 0.09)
Using MK-7 continuously	0.01 (−0.15; 0.17)	0.03 (−0.13; 0.18)	−0.02 (−0.10; 0.07)
Duration of follow-up (months)	0.50 * (0.48; 0.53)	0.50 * (0.48; 0.52)	0.52 * (0.50; 0.54)
**Pre-puberty children (n = 1204)**			
Using MK-7 continuously × Duration of follow-up (months)	0.06 * (0.03; 0.09)	0.10 * (0.07; 0.13)	0.01 (−0.02; 0.05)
Using MK-7 continuously	−0.04 (−0.19; 0.11)	−0.05 (−0.17; 0.07)	0.01 (−0.11; 0.13)
Duration of follow-up (months)	0.54 * (0.52; 0.55)	0.49 * (0.48; 0.51)	0.58 * (0.57; 0.60)
**Total (n = 2298)**			
Using MK-7 continuously × Duration of follow-up (months)	0.05 * (0.02; 0.09)	0.08 * (0.04; 0.12)	0.02 (−0.01; 0.05)
Using MK-7 continuously	−0.02 (−0.20; 0.15)	−0.03 (−0.20; 0.14)	0.00 (−0.13; 0.13)
Duration of follow-up (months)	0.52 * (0.51; 0.54)	0.49 * (0.47; 0.51)	0.57 * (0.55; 0.58)

* *p* < 0.05; Models were adjusted for age (months), gender, baseline BAZ, early sleep, and daily physical activity. Analyses were restricted to baseline and first follow-up assessments because of substantial follow-up imbalance at later visits.

## Data Availability

The original contributions presented in this study are included in the article/Supplementary Material. Further inquiries can be directed to the corresponding author.

## Supplementary Materials

The following supporting information can be downloaded at: https://www.mdpi.com/article/10.3390/nu18121979/s1, Table S1: Duration (months) by follow-up wave (Time) in control and MK-7 groups; Table S2. Height change (mean ± SD) by follow-up duration and baseline characteristics in no-puberty group; Table S3. Height change (mean ± SD) by follow-up duration and baseline characteristics in pre-puberty group.
