# Supplementary material for: Longitudinal Height Growth Patterns Among Children Receiving Menaquinone-7 Supplementation
Source: Nutrients. 2026 Jun 18;18(12):1979. doi: 10.3390/nu18121979 (PMC13304842; doi:10.3390/nu18121979)
Supplement: Supplementary file 1 [file nutrients-18-01979-s001.zip › nutrients-4289248-supplementary.pdf]

Table S1. Duration (months) by follow-up wave (Time) in control and MK-7 groups

Time	Control					Research group				
	Mean ± SD	Median	Min	Max	N	Mean ± SD	Median	Min	Max	N
**0**	0.00 ± 0.00	0	0	0	613	0.00 ± 0.00	0	0	0	537
**1**	8.90 ± 5.50	8	1	28	613	4.97 ± 4.04	4	1	26	535
**2**	11.06 ± 5.55	10	2	25	68	10.05 ± 4.98	9	2	27	525
**3**	12.43 ± 6.50	11	5	25	8	12.23 ± 5.31	11	3	29	258
**4**	—	—	—	—	—	14.29 ± 5.88	14	4	28	150
**5**	—	—	—	—	—	15.05 ± 6.11	14	4	30	73
**6**	—	—	—	—	—	15.07 ± 5.31	15	5	29	43
**7**	—	—	—	—	—	16.41 ± 4.96	17	5	25	27
**8**	—	—	—	—	—	18.47 ± 5.32	20	7	25	17
**9**	—	—	—	—	—	22.56 ± 6.29	25	11	29	9
**10**	—	—	—	—	—	20.00 ± 7.26	20.5	11	28	4
**11**	—	—	—	—	—	19.00 ± 5.00	19	14	24	3
**12**	—	—	—	—	—	19.50 ± 6.36	19.5	15	24	2
**13**	—	—	—	—	—	22.00 ± 5.66	22	18	26	2
**14**	—	—	—	—	—	26.00	26	26	26	1
**15**	—	—	—	—	—	27.00	27	27	27	1
**16**	—	—	—	—	—	27.00	27	27	27	1
**17**	—	—	—	—	—	28.00	28	28	28	1

Table S2. Height change (mean ± SD) by follow-up duration and baseline characteristics in no-puberty group

Variable	Category	Group	1–5 months	6–12 months	>12 months
			Mean (SD)	Mean (SD)	Mean (SD)
Total	,	Control	3.2 ± 1.5	4.7 ± 2.2	8.0 ± 3.0
		Research	2.5 ± 1.6	5.1 ± 2.0	9.2 ± 3.0
		p	<0.001	0.012	0.005
Gender	Female	Control	3.1 ± 1.5	4.7 ± 2.0	7.9 ± 2.8
		Research	2.5 ± 1.7	5.0 ± 1.9	9.3 ± 3.2
		p	0.006	0.083	0.019
	Male	Control	3.5 ± 1.6	4.8 ± 2.7	8.0 ± 3.6
		Research	2.4 ± 1.4	5.3 ± 2.2	9.0 ± 2.6
		p	0.003	0.071	0.141
Stunting	No	Control	3.2 ± 1.5	4.8 ± 2.2	8.1 ± 2.9
		Research	2.5 ± 1.6	5.1 ± 2.0	9.2 ± 3.0
		p	<0.001	0.032	0.016
	Yes	Control	5.0 ± 2.8	2.7 ± 0.6	4.8 ± 1.0
		Research	5.0 ± 0.0	5.8 ± 1.9	8.2 ± 2.5
		p	1.000	0.077	0.050
Thinness	No	Control	3.2 ± 1.5	4.7 ± 2.3	7.9 ± 3.0
		Research	2.5 ± 1.6	5.1 ± 2.0	9.2 ± 3.0
		p	<0.001	0.015	0.003
	Yes	Control	3.3 ± 1.7	5.1 ± 1.8	10.0 ± 2.8
		Research	2.8 ± 1.9	5.5 ± 1.4	8.9 ± 3.7
		p	0.784	0.744	0.464
Overweight/Obesity	No	Control	3.3 ± 1.6	4.8 ± 2.3	7.9 ± 3.1
		Research	2.5 ± 1.6	5.2 ± 2.0	9.0 ± 2.9
		p	<0.001	0.042	0.010
	Yes	Control	2.8 ± 1.1	4.2 ± 1.6	8.4 ± 2.5
		Research	2.4 ± 1.4	4.9 ± 1.8	11.9 ± 2.4
		p	0.429	0.212	0.007
Early sleep	No	Control	3.2 ± 1.5	4.2 ± 1.6	7.9 ± 2.7

Variable	Category	Group	1–5 months	6–12 months	>12 months
			Mean (SD)	Mean (SD)	Mean (SD)
		Research	2.6 ± 1.8	5.1 ± 2.1	8.9 ± 3.3
		p	0.015	0.001	0.341
	Yes	Control	3.3 ± 1.6	5.3 ± 2.6	8.0 ± 3.2
		Research	2.4 ± 1.5	5.1 ± 1.8	9.5 ± 2.8
		p	0.002	0.794	0.004
Physical activity	No	Control	3.1 ± 1.3	4.5 ± 2.2	7.5 ± 2.7
		Research	2.5 ± 1.3	5.1 ± 1.8	8.7 ± 2.8
		p	0.011	0.017	0.050
	Yes	Control	3.4 ± 1.8	5.0 ± 2.3	8.7 ± 3.2
		Research	2.4 ± 1.8	5.2 ± 2.1	9.6 ± 3.1
		p	0.003	0.353	0.146

Table S3. Height change (mean ± SD) by follow-up duration and baseline characteristics in pre-puberty group

Variable	Category	Group	1–5 months	6–12 months	>12 months
			Mean (SD)	Mean (SD)	Mean (SD)
Total	,	Control	3.3 ± 2.0	5.1 ± 2.4	9.1 ± 3.4
		Research	2.8 ± 1.6	6.0 ± 2.3	11.4 ± 3.9
		p	0.021	<0.001	<0.001
Gender	Female	Control	3.2 ± 2.1	5.4 ± 2.6	8.2 ± 3.0
		Research	2.8 ± 1.5	6.1 ± 2.4	11.5 ± 4.3
		p	0.401	0.012	<0.001
	Male	Control	3.4 ± 1.8	5.0 ± 2.2	9.8 ± 3.5
		Research	2.8 ± 1.6	5.9 ± 2.2	11.2 ± 3.6
		p	0.019	0.001	0.005
Stunting	No	Control	3.3 ± 2.0	5.2 ± 2.4	9.2 ± 3.3
		Research	2.8 ± 1.6	6.0 ± 2.2	11.4 ± 4.0
		p	0.019	<0.001	<0.001
	Yes	Control	2.8 ± 1.6	4.6 ± 1.4	8.3 ± 4.4
		Research	2.7 ± 1.1	6.3 ± 3.2	9.0 ± 1.8
		p	0.960	0.321	0.539
Thinness	No	Control	3.3 ± 2.0	5.1 ± 2.5	8.9 ± 3.1
		Research	2.7 ± 1.6	6.0 ± 2.3	11.4 ± 4.0
		p	0.024	<0.001	<0.001
	Yes	Control	3.8 ± 1.5	5.3 ± 1.6	12.4 ± 6.2
		Research	3.4 ± 1.2	6.3 ± 2.4	10.7 ± 3.0
		p	0.842	0.507	0.957
Overweight/Obesity	No	Control	3.3 ± 1.9	5.1 ± 2.4	9.1 ± 3.4
		Research	2.8 ± 1.6	6.0 ± 2.3	11.5 ± 4.0
		p	0.015	<0.001	<0.001
	Yes	Control	3.3 ± 2.5	5.1 ± 3.1	9.4 ± 3.3
		Research	2.8 ± 1.8	5.1 ± 2.0	9.5 ± 3.2
		p	0.779	0.677	0.910
Early sleep	No	Control	3.1 ± 1.8	5.2 ± 2.5	9.7 ± 3.7

Variable	Category	Group	1–5 months	6–12 months	>12 months
			Mean (SD)	Mean (SD)	Mean (SD)
		Research	2.9 ± 1.6	6.4 ± 2.3	11.7 ± 4.2
		p	0.421	<0.001	0.001
	Yes	Control	3.7 ± 2.2	5.0 ± 2.3	8.6 ± 3.0
		Research	2.7 ± 1.5	5.7 ± 2.2	11.1 ± 3.8
		p	0.009	0.014	<0.001
Physical activity	No	Control	3.2 ± 1.8	5.3 ± 2.6	9.3 ± 3.5
		Research	2.8 ± 1.5	6.1 ± 2.5	11.6 ± 4.4
		p	0.092	0.019	<0.001
	Yes	Control	3.5 ± 2.2	5.0 ± 2.2	8.8 ± 3.2
		Research	2.8 ± 1.6	5.9 ± 2.1	11.2 ± 3.7
		p	0.111	<0.001	<0.001
